# Cancer Immunotherapy in Diffuse Large B-Cell Lymphoma

**DOI:** 10.3389/fonc.2018.00351

**Published:** 2018-09-10

**Authors:** Jun Zhang, L. Jeffrey Medeiros, Ken H. Young

**Affiliations:** ^1^Department of Hematopathology, The University of Texas MD Anderson Cancer Center, Houston, TX, United States; ^2^Graduate School of Biomedical Sciences, University of Texas Health Science Center, Houston, TX, United States

**Keywords:** DLBCL, NHL, immunotheray, PD-1, PD-L1, CTLA-4, Chimeric antigen receptor (CAR) T cells therapy, immune checkpoint

## Abstract

Remarkable progress has been made in the field of cancer immunotherapy in the past few years. Immunotherapy has become a standard treatment option for patients with various cancers, including melanoma, lymphoma, and carcinomas of the lungs, kidneys, bladder, and head and neck. Promising immunotherapy approaches, such as chimeric antigen receptor (CAR) T cell therapy and therapeutic blockade of immune checkpoints, in particular cytotoxic T lymphocyte-associated protein 4 (CTLA-4) and programmed cell death protein 1 pathway (PD-1/PD-L1), have boosted the development of new therapeutic regimens for patients with cancer. Immunotherapeutic strategies for diffuse large B-cell lymphoma (DLBCL) include monoclonal anti-CD20 antibody (rituximab), monoclonal anti-PD-1 antibodies (nivolumab and pembrolizumab), monoclonal anti-PD-L1 antibodies (avelumab, durvalumab, and atezolizumab) and chimeric antigen receptor (CAR) T cell therapy. In this review, we outline the latest highlights and progress in using immunotherapy to treat patients with DLBCL, with a focus on the therapeutic blockade of PD-1/PD-L1 and CAR T cell therapy in DLBCL. We also discuss current clinical trials of PD-1/PD-L1 and CAR T cell therapy and review the challenges and opportunities of using immunotherapy for the treatment of DLBCL.

## Introduction

Diffuse large B-cell lymphoma (DLBCL) is the most common type of non-Hodgkin lymphoma. Approximately 60% of DLBCL patients are cured using standard chemotherapy that includes monoclonal anti-CD20 antibody (rituximab), cyclophosphamide, doxorubicin, vincristine, and prednisone (R-CHOP). However, 30–40% of DLBCL patients will develop relapse or have refractory disease that cannot be cured with the standard R-CHOP therapy, indicating the need for more effective therapies for this patient subset. For patients with high-risk DLBCL who often fail R-CHOP therapy, especially patients with high-grade B-cell lymphoma with *MYC* and *BCL2* or *BCL6* translocation, dose-adjusted rituximab, etoposide, prednisone, vincristine, cyclophosphamide, and doxorubicin (DA.R-EPOCH) regimen is a commonly used high intensity regimen.

The development of rituximab was an early step in the application of immunotherapy for the treatment of lymphoma, as it was the first monoclonal antibody (mAb) approved by the US Food and Drug Administration (FDA) for the treatment of patients with advanced stage or relapsed low-grade non-Hodgkin lymphoma, in 1997 (1). See comment in PubMed Commons below Rituximab is a chimeric (mouse and human) monoclonal antibody directed against the B-cell antigen CD20. Rituximab acts via a number of mechanisms including direct antibody dependent cellular cytotoxicity, apoptosis induction, and complement mediated cell death (2). Other monoclonal antibodies that target B-cell antigens, such as CD19 and CD22, also have been developed. CD19 is a specific B cell marker widely expressed during all phases of B cell development until terminal differentiation into plasma cells, with a potential efficacy on a large panel of B cell malignancies. Although initial attempts to target CD19 were unsuccessful, accumulated studies demonstrated targeting CD19 has a therapeutic potential for patients with B cell malignancies (3, 4).

More recently, a number of innovative immunotherapy approaches have shown promising results in patients with relapsed or refractory DLBCL, leading to numerous ongoing clinical trials. CTLA-4 is a negative regulator of T-cell activation, which inhibits anti-tumor immune responses. Blockade of CTLA-4 using the monoclonal antibody ipilimumab improves anti-tumor activity. Ipilimumab was the first immune checkpoint inhibitor approved by the US FDA for the treatment of patients with malignant melanoma. However, the role of the CTLA-4 pathway in DLBCL remains to be elucidated. A phase I clinical trial of ipilimumab in 18 patients with relapsed/refractory B-cell NHL included 3 patients with DLBCL (NCT00089076). Two of these patients had clinical responses and 1 achieved a complete response that lasted more than 31 months. In this study, investigators reported that ipilimumab was well tolerated at the doses used, and that ipilimumab has anti-tumor activity resulting in durable responses in a minority of DLBCL patients (5).

Two highly promising strategies designed to harness the immune system to treat patients with DLBCL are therapeutic blockade of the PD-1/PD-L1 pathway and chimeric antigen receptor (CAR) T cell therapy. These approaches are triggering a paradigm shift in cancer immunotherapy.

## PD-1/PD-L1 signaling pathway

**Figure 1:**
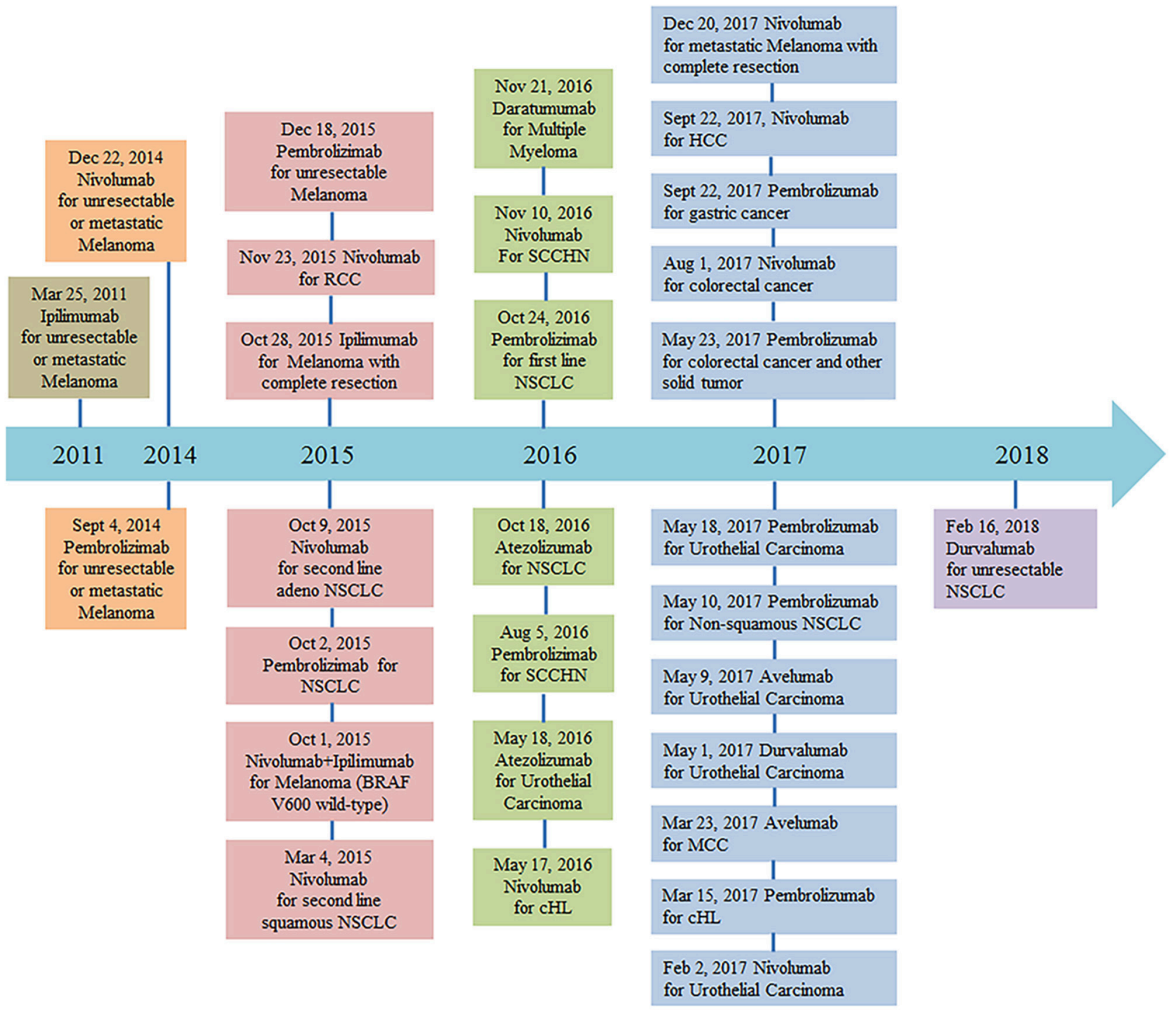
FDA approval timeline of immune checkpoint inhibitors for the treatment of malignancies (https://www.fda.gov/drugs, retrieved Mar 7, 2018). Abbreviations: NSCLC, non–small cell lung cancer; RCC, renal cell carcinoma; cHL, classical Hodgkin Lymphoma; SCCHN, squamous cell carcinoma of the head and neck; MCC, merkel cell carcinoma; HCC, hepatocellular carcinoma.

PD-1/PD-L1 pathway blockade with nivolumab, pembrolizumab, atezolizumab, avelumab, and durvalumab has demonstrated activity in multiple solid tumor malignancies (6–17). Monoclonal anti-PD-1 antibody (nivolumab) was granted designation as a breakthrough therapy for the treatment of patients with relapsed or refractory classical Hodgkin lymphoma on May 17, 2016. The FDA recently granted accelerated approval to another monoclonal anti-PD-1 antibody (pembrolizumab) for the treatment of adult and pediatric patients with refractory primary mediastinal large B-cell lymphoma, or who have relapsed after two or more prior lines of therapy (June 13, 2018). More clinical trials of PD-1 and PD-L1 monoclonal antibodies are currently ongoing (Figure 1). Despite the potential activity of PD-1–blocking antibodies in DLBCL, a subset of patients experiences progressive disease after an initial, often short response (18, 19). Additional research is therefore needed to better understand the reasons for host resistance and to prevent immune-related adverse events.

### Mechanisms of PD-1/PD-L1 signal pathway blockade

**Figure 2:**
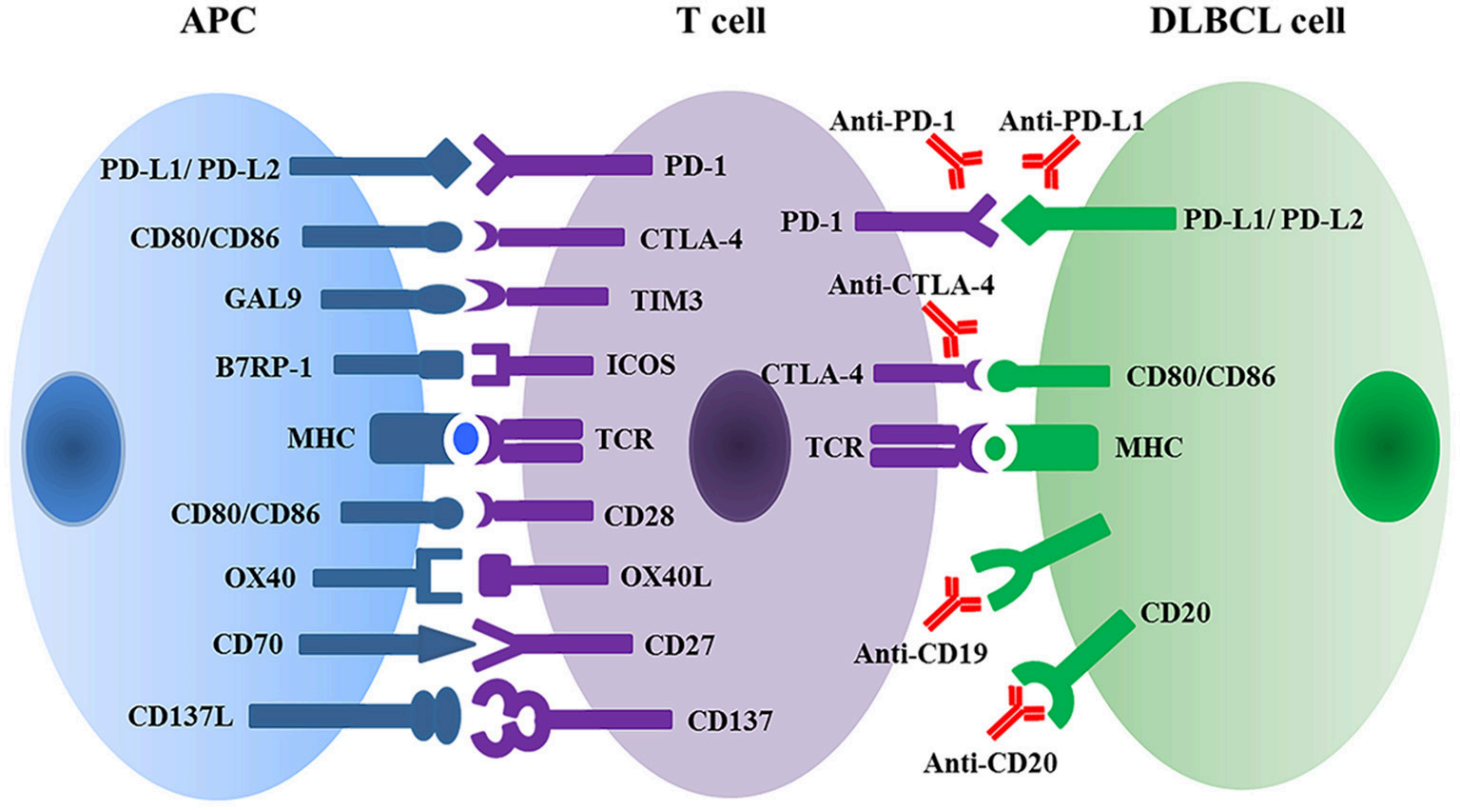
Multiple immune checkpoint and ligand-receptor interactions between T cell and APC or DLBCL malignant cells regulate T cell activation and anti-tumor activity. APC, antigen presenting cell; DLBCL, diffuse large B cell lymphoma; PD1, programmed cell death protein 1; PD-L, programmed cell death ligand; GAL9, galectin 9; TIM3, T cell membrane protein 3; B7RP1, B7-related protein 1; ICOS, inducible T cell co-stimulator; MHC, major histocompatibility complex; TCR, T cell receptor.

The immune system protects the body against illness and infection by bacteria, viruses, fungi, or parasites. Simultaneously, the immune system has the capacity to recognize tumors, inhibit tumor development, and eliminate malignant cells. Cancer cells, however, can evolve and therefore escape from immune surveillance and attack. The mechanisms of cancer immune escape mainly include: reducing the expression of tumor antigens; increasing co-inhibitor expression (e.g., PD-L1, CTLA-4) (20) (Figure 2); secreting suppressive cytokines (e.g., TGF-β and IL-10); and lastly orchestrating an immunosuppressive microenvironment (21, 22).

PD-1 (CD279), a member of the CD28 and CTLA-4 immunoglobulin superfamily, interacts with two B7 family ligands: PD-L1 (CD274 and also known as B7-H1) and PD-L2 (CD273 and also known as B7-DC). PD-1 is expressed on the surface of activated T cells, B cells, natural killer cells, and macrophages as well as by a large proportion of tumor infiltrating lymphocytes (TILs) (15). PD-1 exerts an important immune checkpoint function in the regulation of T-cell mediated immune responses. PD-1 delivers inhibitory signals that regulate T-cell activation, exhaustion, and tolerance through binding to its ligands PD-L1 and PD-L2. PD-L1 and PD-L2 have distinct patterns of expression (23). PD-L1 is expressed primarily by antigen-presenting cells (APC), as well as by a variety of non-hematopoietic cells and tumor cells. PD-L1 expression is induced by pro-inflammatory cytokines, including type I and type II interferons, tumor necrosis factor α (TNF-α) and vascular endothelial growth factor (VEGF) (24, 25). PD-L2 is expressed primarily by dendritic cells and macrophages, and is induced by IL-4 and granulocyte-macrophage colony-stimulating factor (GM-CSF) (26).

In addition to PD-1, PD-L1 also interacts with CD80 expressed on T cells and inhibits T cell responses, whereas PD-L2 also binds to a novel partner repulsive guidance molecule b (RGMb), and plays an important role in pulmonary tolerance (27). Further investigation is needed to explore how these novel pathways are involved in anti-tumor immune responses.

Negative regulation of the PD-1 pathway may be accomplished via multiple mechanisms. The engagement of PD-1 with PD-L1/PD-L2 may suppress T cell activation by competing directly with CD28 for CD80/CD86 binding, resulting in impaired T cell activation and decreased IL-2 production (28). PD-1 binding to PD-L1/PD-L2 results in tyrosine phosphorylation of the PD-1 cytoplasmic regions ITIM and ITSM, which bind the phosphatases SHP-1 and SHP-2, leading to decreased T cell activation and cytokine production (29). PD-1 signaling also inhibits CD28-mediated activation of phosphatidylinositol 3-kinase (PI3K), leading to decreased activation of Akt and reduced expression of transcription factors associated with cell effector functions including GATA3, T-bet, and Eomes (30). Signaling through PD-1 decreases tyrosine phosphorylation of the TCR ζ chain and ZAP-70 (31). PD-1 signaling inhibits the expression of transcription factors associated with effector cell functions, including GATA-3, T-bet, and Eomes (32).

### Clinical immunotherapy of PD-1/PD-L1 inhibitors in DLBCL

As already mentioned, 30–40% of DLBCL patients fail standard therapy and have relapsed or refractory disease (33). PD-1 and PD-L1 expression are not usually a striking feature of patients with cancer (34–36), although several studies have reported over-expression of PD-L1 in specific lymphoma subsets (37, 38). Immune blockade of the PD-1/PD-L1 interaction by monoclonal antibodies can restore the antitumor activity of cytotoxic T cells. Immunotherapy using PD-1/PD-L1 inhibitors has become a clinically validated treatment and has produced durable objective responses and improved overall survival (OS) in patients with solid and hematologic neoplasms. Several monoclonal antibodies targeting the PD-1/PD-L1 pathway are currently in early clinical development including two anti-PD-1 antibodies (nivolumab and pembrolizumab) (Table 1), and three anti-PD-L1 antibodies (avelumab, durvalumab, and atezolizumab) (Table 2).

**Table 1:** Ongoing PD-1 inhibitors trials in DLBCL.

**PD-1 inhibitor**	**Trial name**	**Status**	**Phase**	**Intervention/treatment**	**Immunological target**
Nivolumab	NCT03305445	Not yet recruiting	I/II	Nivolumab, Ipilimumab	PD-1, CTLA-4
	NCT03259529	Recruiting	I/II	Nivolumab, Rituximab, Bendamustine hydrochloride, Gemcitabine	PD-1, CD20
	NCT02038933	Active, not recruiting	II	Nivolumab	PD-1
	NCT03311958	Not yet recruiting	I	Nivolumab	PD-1
	NCT03038672	Not yet recruiting	II	Nivolumab, Varlilumab	PD-1, CD27
	NCT02327078	Recruiting	I/II	Nivolumab, Epacadostat	PD-1
	NCT03015896	Recruiting	I/II	Nivolumab, Lenalidomide	PD-1
Pembrolizumab	NCT03340766	Not yet recruiting	I	Pembrolizumab, Blinatumomab	PD-1, CD19,CD3
	NCT03349450	Not yet recruiting	II	Pembrolizumab, DPX-Survivac, Cyclophosphamide	PD-1
	NCT02362997	Recruiting	II	Pembrolizumab	PD-1
	NCT03401853	Not yet recruiting	II	Pembrolizumab, Rituximab	PD-1, CD20
	NCT03255018	Recruiting	II	Pembrolizumab	PD-1
	NCT03150329	Recruiting	I	Pembrolizumab, Vorinostat	PD-1
	NCT02541565	Recruiting	I	Pembrolizumab, Rituximab, Cyclophosphamide, Doxorubicin Hydrochloride, Prednisone, Vincristine Sulfate	PD-1, CD20
	NCT02650999	Recruiting	I/II	Pembrolizumab	PD-1
	NCT03287817	Recruiting	I/II	Pembrolizumab, AUTO3	PD-1, CD19/22
	NCT03309878	Not yet recruiting	I/II	Pembrolizumab, Mogamulizumab	PD-1, CCR4
	NCT02178722	Recruiting	I/II	Pembrolizumab, INCB024360	PD-1
	NCT02950220	Recruiting	I	Pembrolizumab, Ibrutinib	PD-1
	NCT01953692	Active, not recruiting	I	Pembrolizumab, Lenalidomide	PD-1
	NCT03035331	Recruiting	I/II	Pembrolizumab, Dendritic Cell Therapy	PD-1
	NCT02446457	Active, not recruiting	II	Pembrolizumab, Rituximab, Lenalidomide	PD-1, CD20
	NCT02362035	Active, not recruiting	I/II	Pembrolizumab, Acalabrutinib	PD-1

**Table 2:** Ongoing PD-L1 inhibitors trials in DLBCL.

**PD-L1 inhibitor**	**Trial name**	**Status**	**Phase**	**Intervention/treatment**	**Immunological target**
Atezolizumab	NCT02926833	Recruiting	I/II	Atezolizumab, Axicabtagene Ciloleucel	PD-L1
	NCT03422523	Not yet recruiting	II	Atezolizumab, Rituximab, Gemcitabine, Oxaliplatin	PD-L1, CD20
	NCT02596971	Active, not recruiting	I	Atezolizumab, Obinutuzumab, Rituximab, Bendamustine, Cyclophosphamide, Doxorubicin, Prednisone, Vincristine	PD-L1, CD20
	NCT03321643	Not yet recruiting	I	Atezolizumab, Rituximab, Gemcitabine, Oxaliplatin	PD-L1, CD20
	NCT02729896	Recruiting	I	Atezolizumab, Obinuzumab, Rituximab, PolatuzumabVedotin	PD-L1, CD20, CD79b
	NCT02220842	Recruiting	I	Atezolizumab, Obinutuzumab, Tazemetostat	PD-L1, CD20
	NCT03276468	Not yet recruiting	II	Atezolizumab, Obinutuzumab, Venetoclax	PD-L1, CD20
Durvalumab	NCT02549651	Recruiting	I	Durvalumab, Tremelimumab, AZD9150	PD-L1, CTLA-4
	NCT03212807	Not yet recruiting	II	Durvalumab, Lenalidomide	PD-L1
	NCT03241017	Not yet recruiting	II	Durvalumab	PD-L1
	NCT03003520	Recruiting	II	Durvalumab, Rituximab, Doxorubicin, Vincristine, Cyclophosphamide, Prednisone, Lenalidomide	PD-L1, CD20
	NCT02401048	Active, not recruiting	I/II	Durvalumab Ibrutinib	PD-L1
	NCT02706405	Recruiting	I	Durvalumab Autologous Anti-CD19CAR-4-1BB-CD3zeta-EGFRt-expressing CD4+/CD8+ Central Memory T-lymphocytes JCAR014, Cyclophosphamide, Fludarabine Phosphate	PD-L1
	NCT02205333	Terminated	I/II	Durvalumab, MEDI6469, Rituximab, Tremelimumab	PD-L1, OX40, CD20, CTLA-4
Avelumab	NCT03244176	Recruiting	I	Avelumab	PD-L1
	NCT02951156	Recruiting	III	Avelumab, Utomilumab, Rituximab, Azacitidine, Bendamustine, Gemcitabine, Oxaliplatin	PD-L1, 4-1BB, CD20
	NCT03440567	Not yet recruiting	I	Avelumab, Utomilumab, Rituximab, Ibrutinib, Carboplatin, Etoposide Phosphate, Ifosfamide	PD-L1, 4-1BB, CD20

Nivolumab is a human IgG4 anti-PD-1 monoclonal antibody. Multiple phase I/II studies of nivolumab are evaluating (or planning to evaluate) its efficacy in combination with agents such as ipilimumab (NCT03305445), rituximab and chemotherapy (NCT03259529), varlilumab (anti-CD27) (NCT03038672), the IDO1 inhibitor epacadostat (NCT02327078), and lenalidomide (NCT03015896) in participants with DLBCL.

Although early results in phase I studies were promising, only one phase II study has been reported for the use of PD-1/PD-L1 inhibitors in DLBCL patients, “A single-arm, open-label, phase 2 study of nivolumab (BMS-936558) in subjects with relapsed or refractory DLBCL after failure of autologous stem cell transplant (ASCT) or after failure of at least two prior multi-agent chemotherapy regimens in subjects who are not candidates for ASCT”. In this study, 161 participants were enrolled and 121 participants entered the treatment period. Participants were enrolled, but not treated due to adverse events (*n* = 2), withdrawal of consent (*n* = 2), death (*n* = 2), or they no longer met study criteria (*n* = 34). Finally, 102 participants completed the treatment period. Nivolumab 3 mg/kg administered as an IV infusion on treatment day 1 of each 14 day cycle until disease progression or discontinuation due to toxicity, withdrawal of study consent, or the study ends. Nivolumab therapy resulted in an overall response rate (ORR) of 10.3% in the ASCT-failed group (complete response [CR], 3.4%; partial response [PR], 6.9%) and 2.9% in the ASCT ineligible group (CR, 0%; PR, 2.9%). The median duration of response was 11.4 months in the ASCT-failed group and 8.3 months in the ASCT-ineligible group (NCT02038933).

A phase I trial of nivolumab monotherapy recruited patients with heavily pretreated relapsed or refractory lymphoid malignancies including 11 patients with DLBCL. Four (36%) patients responded (2 CR and 2 PR). The median follow-up duration for patients with DLBCL was 22.7 weeks; 1 of 4 patients with DLBCL has had an ongoing response, and 2 patients continue to be followed (18).

Pembrolizumab is another humanized IgG4 anti-PD-1 monoclonal antibody. Various phase I/II studies of the PD-1 antibody pembrolizumab are still ongoing, either as a single agent or in combination with antibodies, small molecular inhibitors, immunotherapeutic vaccine, dendritic cell therapy, and CAR T cell treatment in participants with DLBCL.

Atezolizumab is a human IgG1 monoclonal antibody that targets PD-L1. Seven phase I/II studies of atezolizumab are ongoing to evaluate its efficacy in combination with other agents such as CAR T cells, antibodies, small molecular inhibitors, and chemotherapy in participants with DLBCL.

Durvalumab is a human IgG1 monoclonal antibody that targets PD-L1. One phase II clinical trial of durvalumab as a single agent is ongoing to assess the progression-free survival (PFS) two years after ASCT in high-risk DLBCL patients. Five phase I/II studies are underway to evaluate the efficacy of durvalumab in combination with antibodies, small molecular inhibitors, and chemotherapy as well as CAR T cell therapy in participants with DLBCL. Another phase II clinical trial of durvalumab in combination with monoclonal antibodies directed against CD20, OX40 and CTLA4 designed to determine the optimal dose of MEDI6469 (anti-OX40) that is safe and tolerable in participants with DLBCL was terminated early at the sponsor's discretion due technical problems (NCT02205333).

Another human IgG1 anti-PD-L1 monoclonal antibody is avelumab. An early phase I study of avelumab as a single agent is ongoing to evaluate the feasibility of adding induction and maintenance avelumab to standard R-CHOP therapy in patients with stage II, III, and IV DLBCL (NCT03244176). An ongoing phase III study is evaluating the efficacy of avelumab in combination with a variety of agents for relapsed or refractory DLBCL patients; these agents include utomilumab (anti-4-1BB/CD137), rituximab, azacitidine, bendamustine, gemcitabine, and oxaliplatin, (NCT02951156). A recent phase I trial is studying the side effects and optimal dosing of avelumab, utomilumab, rituximab, ibrutinib, and combination chemotherapy for treating patients with DLBCL or relapsed/refractory mantle cell lymphoma, but is not yet recruiting (NCT03440567).

Pidilizumab (MDV9300, Medivation, Inc) was originally considered a monoclonal antibody binding to PD-1. This agent yielded encouraging results in phase II clinical trials for DLBCL. However, recent evidence suggests that PD-1 is not the target of pidilizumab. The FDA has lifted its partial clinical hold on the investigational new drug (IND) application for pidilizumab (MDV9300) in hematological malignancies and has confirmed that the phase II clinical trial in patients with relapsed or refractory DLBCL, as well as other studies that cross reference the IND, may now proceed. The partial clinical hold was not related to any safety concerns. The investigator brochure, protocols, and informed consent documents related to the phase II trial have satisfactorily been revised to reflect that the manufacturer's understands that PD-1 is not the target of pidilizumab. No patients had yet been enrolled in the trial which commenced in late 2015. Patients who were receiving pidilizumab through investigator-sponsored trials have continued to receive treatment and the investigators have been informed to update their protocols and informed consent documents to state that pidilizumab is not an anti-PD-1 antibody, but an anti-Delta-like ligand 1 antibody (39, 40).

Immune checkpoint blockade has promising potential in DLBCL therapy. A subgroup of patients with advanced cancers may respond to single-agent immune checkpoint blockade, however, most patients do not respond to monotherapy (41). In order to enhance the antitumor efficacy, a combination of multiple therapeutic approaches is urgently needed. Many clinical trials are ongoing to evaluate the synergistic efficacy of immune checkpoint inhibitors in combination with other agents, which mainly includes co-inhibitory blockade (anti-CTLA-4), co-stimulatory agonists (anti-OX40, anti-4-1BB), rituximab (anti-CD20) and conventional chemotherapy. Both PD-1 and CTLA-4 are expressed on T cells, but they play different regulatory functions via different signaling pathways in suppressing T cell activation and proliferation. The combined therapy of anti-PD-1 and anti-CTLA-4 has demonstrated synergistic efficacy and improve antitumor activities. In contrast, both OX40 and 4-1BB are members of the tumor necrosis factor (TNF) family of co-stimulatory receptors, expressed on the surface of CD4^+^ and CD8^+^ T cells. Agonist antibodies anti-OX40 and anti-4-1BB promote T cell activation, growth, and survival and enhance antitumor functions. Conventional chemotherapy in combination with immune checkpoint blockade has shown synergistic efficacy by releasing multiple tumor neoantigens or modifying the tumor microenvironment (Tables 1, 2).

### Challenges and opportunities for blocking the PD-1/PD-L1 pathway

Targeting the PD-1/PD-L1 pathway in patients with DLBCL is a promising treatment strategy. However, there are adverse events associated with PD-1/PD-L1 inhibitors that reflect the actions of the PD-1 pathway in the regulation of immune responses. PD-1 pathway blockade can cause immune-related adverse events that may affect almost all tissues. Toxicities related to immune checkpoint inhibitors typically include dermatologic manifestations, diarrhea, colitis, hepatotoxicity, endocrinopathies, and pneumonitis (42–44). Based on the experience of immune-checkpoint inhibitors in patients with solid tumors, the occurrence of grade 3–4 immune-related adverse events is approximately 20% with ipilimumab, compared with 5–10% with nivolumab or pembrolizumab (45). Generally, PD-1 pathway blockade is associated with fewer and less severe toxicities compared with CTLA-4 blockade. Toxicities can be managed with immune-modulating agents including corticosteroids and infliximab. Early studies suggest that combination therapy with CTLA-4 and PD-1 inhibitors may increase efficacy, but at the cost of increased toxicity (46). However, the combination of anti-CTLA-4 and anti-PD-1 antibodies demonstrated a similar safety and efficacy profile compared to a previous report for anti-PD-1 monotherapy in Hodgkin lymphoma, non-Hodgkin lymphoma (NHL), and multiple myeloma (19).

In patients with NHL, severe immune-related adverse events have been rare to date. A phase I trial of ipilimumab in patients with relapsed/refractory B-cell lymphoma is designed to evaluate safety, immunologic activity, and potential clinical efficacy. Diarrhea has been reported frequently among patients receiving ipilimumab, in 56%, with 28% of these patients developing grade 3–4 adverse events (5). Among patients with relapsed NHL receiving nivolumab within a phase Ib trial, 4% developed grade 3–5 pneumonitis (18). Another adverse event is fatigue, reported to occur in 13–56% of patients, mostly grade 1–2 (5). In clinical practice, adverse events associated with nivolumabhave been well tolerated and this agent has exhibited antitumor activity in extensively pretreated patients with relapsed or refractory B- and T-cell lymphomas (5, 18).

Biomarker data might be useful in guiding dose and regimen selection in early clinical development. However, a correlation between the expression of PD-L1 by DLBCL cells and response to PD-1 inhibitors has not been confirmed and remains controversial (39). Evaluation of PD-L1 expression by tumor-cells as a predictive marker has been inconclusive. This observation might be due to complex dynamics of expression depending on the tumor microenvironment and the lack of standardized immunohistochemical assessment of PD-L1 expression (47).

## CAR T cell therapy

CAR T-cells are autologous, polyclonal T lymphocytes genetically engineered to express a tumor-targeting receptor, directing the T cells to bind to a specific tumor-associated antigen. CAR T cells are composed of an extracellular single chain variable fragment (scFv) and intracellular signaling domains that allow T cells to effect functions independent of major histocompatibility complex (MHC) antigens. Depending on differences in the intracellular signaling domains and cytokine secretion, CAR T cells have been classified as first-, second-, third- and fourth-generation. First-generation CAR T cells consisted of an extracellular scFv and a single intracellular signaling domain CD3ζ. The limited activity of this generation was probably attributable to their inability to adequately activate T cells, especially in cases where tumor cells did not express T cell co-stimulatory molecules (48). Subsequently, second (and third and fourth)-generation CAR T cells included co-stimulatory domains, such as CD28 or CD137 (4-1BB), to improve expansion and persistence of T cells (49, 50). Kochenderfer first reported the anti-tumor efficacy of an anti-CD19 CAR T cell containing the CD28 costimulatory domain in aggressive lymphoma (51). In order to enhance the activation of CAR T cells, third-generation CAR T cells were designed by combining two signaling domains among CD28, CD27, 4-1BB, ICOS, and OX40 (52–56). Including two co-stimulatory domains into CAR T cells can improve the tumor cell-killing efficacy. However, because of the activation of multiple intracellular signaling caused by the co-stimulatory domains of third-generation CAR T cells, abundant cytokines might be released which may result in a life-threatening cytokine storm (57). In order to enhance their tumor cell-killing efficacy and impact local suppressive cells, fourth-generation CAR T cells were engineered with an inducible expression component, such as cytokine IL-12, and also are known as T cells redirected for universal cytokine-mediated killing (TRUCKs). TRUCKs not only increase the activation of CAR T cells, they also induce cytokines and attract innate immune cells to eliminate antigen-negative cancer cells (58). In addition, for safety considerations, an inducible caspase 9 self-withdrawal genetic design allows for rapid elimination of infused CAR T cells once the anti-tumor mission is accomplished (59, 60).

### Clinical trials of CAR T cells as therapy in DLBCL

CAR T cell therapies have been most efficacious in patients with B-cell acute lymphoblastic leukemia; less data are available for patients with DLBCL. According to the American Cancer Society, ~72,000 children and adults in the US will be diagnosed with non-Hodgkin lymphoma in 2017; 60% of these cases are aggressive neoplasms with the most common type being DLBCL. The typical survival duration of patients with DLBCL who have disease progression after chemotherapy or ASCT is 9 months. The cumulative promising data indicate that immunotherapy using CAR T cells offers hope for achieving long-term survival in patients with relapse/refractory DLBCL or follicular lymphoma (FL).

Investigators from Kite Pharma developed a clinical trial of CD19-CAR T cells (NCT02348216) that was approved on October 2017, becoming the first CAR T therapy approved by the FDA for the treatment of adults with relapsed or refractory DLBCL after two or more lines of systemic therapy. CD19-targeting CAR T cell therapy showed that 42% of patients with refractory DLBCL remained in remission at 15 months following treatment with axi-cel (marketed as Yescarta). Axi-cel CAR T cell therapy is the second gene therapy approved by the FDA and the first for adult patients with DLBCL after failing at least two other kinds of treatment; the types of large B-cell lymphoma in this study include DLBCL not otherwise specified (NOS), primary mediastinal large B-cell lymphoma, DLBCL arising from follicular lymphoma, and cases that fit into the new World Health organization category of high grade B-cell lymphoma (e.g., DLBCL with double hit genetics). This study, named ZUMA-1, also reported measurable responses in 82% of patients and complete responses in 54%. Over half (56%) of patients were alive at 15 months following therapy, with some remaining cancer-free for 2 years post-treatment. Among the 111 patients who were enrolled, axi-cel was successfully manufactured for 110 and administered to 101. The median age of these patients was 58 years (range, 23–76 years). Most (85%) patients in the study group had stage III or IV disease; 77% had disease that was resistant to second line or subsequent therapies, 21% had disease relapse after transplantation, 69% had received at least three previous therapies, and 26% had a history of primary refractory disease. Among the 101 patients who received axi-cel, the ORR was 82%, with a 54% CR. With a median follow-up of 15.4 months, 42% of patients continued to have a response, with 40% in CR. The overall rate of survival at 18 months was 52%. The most common adverse events of grade 3 or higher during treatment were neutropenia (78% of the patients), anemia (43%), and thrombocytopenia (38%). Grade 3 or higher cytokine release syndrome (CRS) and neurologic events occurred in 13 and 28% of patients, respectively. Three patients died during treatment. In this multicenter study, patients with refractory DLBCL who received CAR T-cell therapy with axi-cel had high levels of durable response, with a safety profile that included myelosuppression, CRS, and neurologic events (61).

Kochenderfer and colleagues at the National Cancer Institute were the first to report a partial response (PR) lasting 32 weeks after infusing autologous T cells directed against CD19 in a patient with FL (62). This group later published seven patients with DLBCL: four patients achieved a CR, two achieved a PR, and one had stable disease (SD) (57). Recently, Kochenderfer et al. reported results for 22 patients with advanced-stage lymphoma in a clinical trial of CAR-19 T cells preceded by low-dose chemotherapy, including 19 patients with DLBCL, two patients with FL, and one patient with mantle cell lymphoma. Patients received a single dose of CAR-19 T cells 2 days after a low-dose chemotherapy conditioning regimen of cyclophosphamide plus fludarabine. This study showed that CAR-19 T cells are an effective therapy for lymphoma patients and with lower doses of chemotherapy than they previously used; the ORR was 73%, with 55% achieving CR and 18% achieving PR. Eleven of 12 patients remain in CR and grade 3 or 4 neurologic toxicities in about half of the patients resolved completely (51).

Investigators from the University of Pennsylvania Medical Center have collaborated with Novartis to develop a second-generation CD19-CAR T cell named CTL019. This CAR consists of a murine anti-CD19 scFv, a CD8 hinge, a trans-membrane domain, 4-1BB (co-stimulatory molecule), and CD3ζ. This group has conducted a phase IIa clinical trial of CTL019 cells in patients with relapsed or refractory CD19+ non-Hodgkin lymphomas (NCT02030834); 29 patients (19 DLBCL; 8 FL; 2 MCL) enrolled and 20 patients received CTL019 per protocol dose (12 DLBCL; 7 FL; 1 MCL). Pre-infusion chemotherapy regimens were EPOCH (*n* = 2); cyclophosphamide (*n* = 9); radiation + cyclophosphamide (*n* = 2); bendamustine (*n* = 6); cyclophosphamide-fludarabine (*n* = 1). Cytokine release syndrome occurred in 15 patients (13 grade 2; 2 grade 3). Neurologic toxicity occurred in 3 patients: transient delirium (1 grade 2, 1 grade 3) and 1 possibly related, grade 5 encephalopathy. For 18 patients evaluable for response at 3 months (12 DLBCL; 6 FL), the ORR was 67% (DLBCL 50%; FL 100%). At a median follow up 6 months, progression-free survival for evaluable patients was 59% (DLBCL 37%; FL 100%). This report shows that CTL019 cells induce durable responses in patients with relapsed/refractory DLBCL and FL with acceptable toxicity (63).

Recently, interim results from a global, pivotal multi-center phase II JULIET trial (NCT02445248) of CTL019 (tisagenlecleucel) showed durable complete responses in adults with relapsed/refractory DLBCL. The ORR at 3 months was 45% (23 of 51 patients evaluated), with 37% achieving CR and 8% achieving PR. The patients with CR remained stable from 3 months through data cutoff among the study cohort (64).

Investigators from the Fred Hutchinson Cancer Research Center, Memorial Sloan Kettering Cancer Center, and Seattle Children's Research Institute have collaborated with Juno Therapeutics to conduct several clinical trials of CD19-CAR T cell products: JCAR014, JCAR015, JCAR017, JCAR021, and others. Among them, updated results from the ongoing TRANSCEND study of JCAR017, which contains the 4-1BB costimulatory domain, in patients with relapsed or refractory aggressive non-Hodgkin lymphoma were presented during 2017 American Society of Hematology meeting. The core group (*n* = 49) included patients with DLBCL (NOS and transformed from follicular lymphoma) who were ECOG performance status 0–1. These patients represented a highly refractory population based on factors associated with a poor prognosis, including older patient age, double, or triple hit genetics (*MYC* and *BCL2* and/or *BCL6* rearrangement), and the DLBCL being refractory to chemotherapy. Dose level 1 (DL1 = 50 million cells) showed a 3 month ORR of 52% (11/21 patients) and a 3 month CR rate of 33% (7/21). Dose level 2 (DL2 = 100 million cells), the dose in the pivotal cohort of the TRANSCEND study, showed a 3 month overall response rate (ORR) of 80% (12/15) and a 3 month complete response (CR) rate of 73% (11/15) in the core group. These data support a dose response relationship. Across both doses in the core group, the best overall response was 84% (41/49 patients) and the best overall CR rate was 61% (30/49). There was no increase in CRS or neurotoxicity (NT) rates associated with the higher dose or between the full and core groups. Across doses in the full group, 1 of 69 (1%) patients experienced severe CRS and 10 (14%) patients experienced severe NT. Twenty-one of 69 (30%) patients had any grade CRS and 14 (20%) patients had any grade NT. 64% (44/69) of patients had no evidence of CRS or NT. The most common treatment-emergent adverse events other than CRS and NT that occurred at ≥25% in the full group included neutropenia (41%), fatigue (30%), thrombocytopenia (30%), and anemia (26%) (65–67).

### Challenges and opportunities for CAR T cell therapy

CAR T cells have shown promising efficacy in patients with DLBCL, including those with relapsed or refractory DLBCL. However, this therapy can be associated with unexpected toxicities that can be life-threatening, including CRS, NT, and “on-target off-tumor” recognition. The challenges are to reduce toxicity, prolong disease-free survival, and to determine which factors can predict relapse of DLBCL after successful CAR T cell therapy.

Cytokine release syndrome is a systemic inflammatory response to the activation and proliferation of CAR T cells. The clinical features of CRS include high fever, fatigue, nausea, malaise, hypotension, cardiac dysfunction, renal impairment, hepatic failure, capillary leak, and disseminated intravascular coagulation (68). CRS is associated with a dramatic elevation of inflammatory cytokines in the serum including C reactive protein (CRP), interferon-γ, ferritin, granulocyte macrophage colony-stimulating factor, IL-10, and IL-6 following CAR T-cell infusion (69–72). CRS occurs most frequently within the first 2 weeks after CAR T cell infusion. Clinical management schemes of CRS include administration of steroids and the IL-6 receptor blocking antibody, tocilizumab (68, 73). However, steroids blunt the anti-tumor function of CAR T cells and the long-term impact of tocilizumab on CAR T cell function remains unclear. It remains a challenge to control CRS without inhibiting the anti-tumor efficacy of CAR T cell therapy.

Neurologic adverse events have been observed in many patients receiving CD19-CAR T cell therapy. Reversible symptoms of NT, including confusion, delirium, expressive aphasia, encephalopathy, and seizures, have been reported in several studies (51, 69, 74–77). In some patients, CD19-CAR T cells have been found in cerebrospinal fluid (74, 76). Whether neurological toxicities are solely restricted to CD19-specific CAR T cells or are associated generally with CAR T cell therapy remains unclear and the potential causes of NT remain to be elucidated. The postulated pathophysiological mechanisms include cytokine diffusion and/or translocation of activated CAR T cells across the blood brain barrier.

On-target off-tumor recognition side effects caused by depletion of healthy CD19-positive B-cells by CAR T cells are also an issue. B cell aplasia is a common adverse event in CAR T cells trials targeting B cell malignancies (75, 77, 78). Off-tumor recognition side effects in CAR T cell treated patients also can occur as a result of cross-reactivity of the engineered antigen binding domain with a non-related surface protein.

Selective depletion of CAR T cells can be approached by the use of “self-withdrawal CARs” in which is inserted an inducible caspase 9 (ICasp9) (79, 80). Current T-cell engineering approaches redirect patient T cells to tumors by transducing them with antigen-specific T-cell receptors (TCRs) or CARs that target a single antigen. However, healthy tissues that express the targeted antigen may undergo CAR T cell-mediated damage. A novel strategy that combines antigen recognition with balanced signaling promotes selective tumor eradication by engineered T cells (81). In trials using CD19-targeting T-cells, CD19-negative clones have expanded and caused progressive disease (82). The approach of increasing the specificity of CARs is to combine more CAR T cells to recognize multiple targets. This treatment strategy may help broaden the applicability and avoid some of the side effects of targeted T-cell therapies. In addition, a novel agent that blocks IL-35 may support CAR T cell therapy by reducing the inhibitory effect of regulatory T cells that may be of value in the future (83). Furthermore, several small molecule inhibitors, such as ibrutinib (Bruton tyrosine kinase inhibitor) (84), ABT-199 (Bcl-2 inhibitor) (85), and JQ-1 (bromodomain inhibitor) (86), has shown impressive potential for treating DLBCL patients. CAR T immunotherapy in combination with a small molecule inhibitor is likely to provide greater benefit for the treatment of patients with DLBCL.

## Conclusions

Cancer immunotherapy that harnesses the host immune system in novel ways to kill tumor cells is emerging. Immunotherapy offers promising opportunities with the potential to induce sustained remissions, and is expected to become a “game changer” for the treatment of patients with cancer. Novel immunotherapy regimens, PD-1/PD-L1, and CTLA-4 checkpoint inhibitors, and CAR T cells have shown promising potential in the treatment of patients with DLBCL.

Early clinical trials using PD-1/PD-L1 checkpoint inhibitors including two anti-PD-1 antibodies (nivolumab and pembrolizumab), and three anti-PD-L1 antibodies (avelumab, durvalumab, and atezolizumab), have shown great promise. CAR T cell therapy also has shown remarkable activity in patients with refractory DLBCL. Yescarta, a CAR T cell immunotherapy, has been approved by the FDA for use in adults with large B-cell lymphoma after at least two other kinds of treatment have failed. Numerous ongoing clinical trials will undoubtedly offer the hope of achieving long-term survival in patients with relapsed or refractory disease.

## Author contributions

All authors listed have made a substantial, direct and intellectual contribution to the work, and approved it for publication.

### Conflict of interest statement

The authors declare that the research was conducted in the absence of any commercial or financial relationships that could be construed as a potential conflict of interest.
